# ESOPHAGOCELE DUE TO TWO TIMES CAUSTIC INGESTIONS: RESECTION THROUGH VIDEOTHORACOSCOPY

**DOI:** 10.1590/0102-672020220002e1705

**Published:** 2023-01-09

**Authors:** Iuri Pedreira Filardi ALVES, Valdir TERCIOTI, João de Souza COELHO, José Antonio Possatto FERRER, Nelson Adami ANDREOLLO, Luiz Roberto LOPES

**Affiliations:** 1 Universidade Estadual de Campinas, Faculty of Medical Sciences, Department of Surgery and Gastrocenter, Digestive Diseases Surgical Unit – Campinas (SP), Brazil.

**Keywords:** Caustics, Esophageal Stenosis, Esophagoplasty, Mucocele, Esophagectomy, Thoracoscopy, Cáusticos, Estenose Esofágica, Esofagoplastia, Mucocele, Esofagectomia, Toracoscopia

## Abstract

**BACKGROUND:**

Caustic ingestion is still a health problem of utmost importance in the West. In developing countries, this incident remains at increase and it is associated with unfavorable factors like social, economic, and educational handicaps, besides a lack of prevention. Esophagocele is a rare consequence of caustic ingestion.

**AIM:**

We aimed to describe a patient with multiple caustic ingestions who presented an esophagocele resected by videothoracoscopy.

**METHODS:**

A woman ingested caustic soda when she was only 17 years old in a suicidal attempt during a depressive crisis. Initially, she was submitted to a retrosternal esophagocoloplasty with the maintenance of her damaged esophagus. After 1 year of this first surgery, she ingested caustic soda again in a new suicidal attempt. Her transposed large bowel in the first surgery became narrow, being replaced in a second surgery by a retrosternal esophagogastroplasty. Still, at the second surgery, her damaged esophagus remained in its original position in the posterior mediastinum. However, after 5 years, she developed an esophagocele.

**RESULTS:**

The esophagocele was resected through videothoracoscopy in a prone position, employing four trocars. The postoperative was uneventful.

**CONCLUSION:**

Esophageal exclusion must always be recorded because esophagocele presents unspecific symptoms. The videothoracoscopy in a prone position is an excellent technical option to resect esophagoceles.

## RESUMO

**RACIONAL:**

A ingestão de produtos cáusticos ainda é um problema de saúde de extrema importância no Ocidente. Nos países em desenvolvimento, este incidente continua em ascensão e está associada a fatores desfavoráveis como sociais, econômicos e educacionais, além da falta de prevenção. A esofagocele é uma consequência rara da ingestão de cáusticos.

**OBJETIVO:**

Nosso objetivo é descrever um paciente com múltiplas ingestões cáusticas que apresentou uma esofagocele ressecada por videotoracoscopia.

**MÉTODOS:**

Doente feminina que ingeriu soda cáustica com 17 anos de idade, como tentativa de suicídio, durante uma crise depressiva. Inicialmente, foi submetida a esofagocoloplastia retroesternal com manutenção do esôfago lesado. Após um ano desta primeira cirurgia, voltou a ingerir soda cáustica, em nova tentativa de suicídio. Seu intestino grosso transposto na primeira cirurgia tornou-se estenosado, sendo substituído em uma segunda cirurgia, por esofagogastroplastia retroesternal. Ainda assim, nesta segunda cirurgia, o esôfago lesado permaneceu em sua posição original no mediastino posterior. No entanto, após cinco anos, ela desenvolveu uma esofagocele.

**RESULTADOS:**

A esofagocele foi ressecada por videotoracoscopia, em decúbito ventral, empregando-se quatro trocartes. O pós-operatório transcorreu sem intercorrências.

**CONCLUSÕES:**

A exclusão esofágica deve ser sempre registrada, pois a esofagocele apresenta sintomas inespecíficos. A videotoracoscopia em posição prona é uma excelente opção técnica para ressecção de esofagoceles.

## INTRODUCTION

Caustic ingestion is a health problem of utmost importance in the West because of its impact on the population morbidity and mortality. In developing countries, this disease remains increasing and it is associated with unfavorable factors like social, economic, and educational handicaps, and a lack of prevention. Worldwide, children are the most affected, accounting for about 80% of the cases, mainly by accidental ingestion. On the contrary, the main cause of caustic ingestion in the adult population seems to be secondary to suicidal attempt^1,3^.

Moreover, the impossibility of endoscopic dilation or its failure points out a surgical treatment, aiming to re-establish the alimentary tract through esophagus replacement. This replacement can be achieved by interposition of the retrosternal stomach or by transposing the large bowel, which is actually the preferred method. Resection of a damaged esophagus is optional. The resection of the damaged esophagus increases surgical morbidity-mortality when compared to no resection. However, with increased risk of cancer, lack of possibility of endoscopic surveillance, and risk of infection at a rate of 50% at 5 years of the resultant esophagocele, some authors advise to resect the damaged esophagus^1,3^.

The objective of this study was to report the surgical technique employed in a 17-year-old adolescent who attempted suicide twice by swallowing a moderate quantity of caustic products. Later, she presented an esophagocele.

## METHODS

The patient was referred to our hospital after first caustic ingestion, due to a suicidal attempt, with dysphagia for her own saliva. The upper digestive endoscopy and the contrast radiography of the esophagus showed severe esophageal stenosis. No endoscopic treatment was possible, and she was submitted to retrosternal esophagocoloplasty. The postoperative was uneventful and she was discharged after started eating soft and solid foods. Concomitantly, she started psychiatric treatment, which included follow-up in an outpatient unit.

After 1 year, the patient presented a new depression crisis caused by the interruption of her psychiatric treatment. Again, she swallowed a caustic product in a new suicidal attempt and was admitted with dysphagia which was severe for solid and soft foods and also associated with weight loss. Thus, a new digestive endoscopy and contrast radiography showed complete stenosis of the colon, initiating at the neck level.

Therefore, she was submitted to her second surgery, which consisted of a large bowel resection previously transposed, associated with a new alimentary tract reconstruction employing a gastric tube and esophagogastric anastomosis at the neck, plus jejunostomy for early enteral feeding. Again, her damaged esophagus was not resected at that time, and the postoperative course was uneventful.

She remained well for 5 years after this second surgery described above, with a regular follow-up in an outpatient unit, with good recovery of weight. However, after this period, she complained every day of retrosternal pain, which was worsened by deep inspiration, pain in the left upper quadrant, weight loss, loss of appetite, and fever. The patient signed a consent form, authorizing this report.

## RESULTS

The patient was readmitted to the hospital and CT scans showed an esophagocele associated with an abdominal left sub-diaphragm abscess, which, probably, revealed itself with that esophagocele. This left sub-diaphragm abscess was treated by ultrasound-guided percutaneous drainage and antibiotic therapy, with a resolution of the pain and fever. She was discharged with no symptoms.

**Figure 1.**
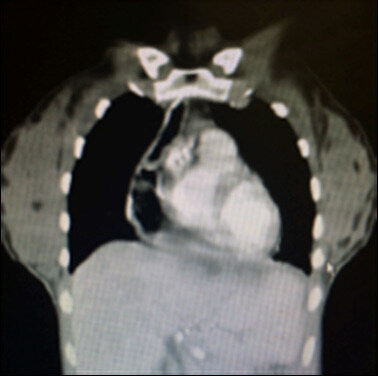
Computed tomography scan showing esophagocele.

**Figure 2.**
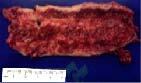
The esophagocele resected in the third surgery by videothoracoscopy.

Six months later, she returned with the same symptoms described above. A new CT scan showed an increased esophagocele (Figure 1). The patient was submitted to the third surgery, which consisted of esophagocele resection through videothoracoscopy in a prone position, employing four trocars (two trocars of 10 mm and two of 5 mm). A thoracic drain was a chest tube that was inserted and removed on the third postoperative day, after the control x-ray, showing complete lung expansion. Figure 2 shows the esophagocele resected.

She had an uneventful postoperative period without vasoactive drugs or blood transfusions, and an oral diet was initiated on the second postoperative day. She was discharged on the fifth postoperative day. At late follow-up in the outpatient unit, she presented weight recovery, with no fever or any dysphagia.

## DISCUSSION

Esophagocele, or esophageal mucocele, is a rare condition with unknown frequency and is not well described in the medical literature^4,5^. Esophageal exclusion may develop an esophagocele, when the proximal and distal esophagus is occluded, as a consequence of the accumulation of secretion in a closed organ^6^. This event rarely brings symptoms, however, it can cause thoracic pain, tracheal compression, fistulization into tracheobronchial tree and neck, thoracic and abdominal abscesses, abdominal pain, cough, vomiting, fever, infection, and sepsis^1,2,4,7^. Most of the esophagoceles are small, probably because in many situations the damaged mucosa suffers atrophy^2,9^.

CT scans and magnetic resonance imaging (MRI) are the main diagnostic tools, which usually show a cystic image in the mediastinum. Most of the symptomatic esophagoceles are treated with thoracotomy followed by esophageal resection^4,6,7^. However, there are case reports of treatment through CT scan-guided drainage^2^. Other case reports mention mucosal ablation with 100% alcohol^2^. In addition, there are reports that include some patients with high surgical risk for thoracotomy and surgical drainage of the distal esophagus through esophagus-jejunum anastomosis with a Roux-en-Y intestinal loop may be an option^4,5^.

Esophagocoloplasty is the choice treatment for caustic stenosis, and many authors do not recommend esophagectomy at the same surgical time^1^. Sometimes, resection and alimentary tract reconstructions are not feasible in the same surgery due to increased morbidity, making esophageal exclusion an option of treatment^7,9^.

Pavankumar et al. reported a case of a 22-year-old female who ingested a chicken bone and presented an esophageal perforation, treated by esophagostomy and feeding jejunostomy. Six weeks later, the patient underwent laparoscopic-assisted retrosternal gastric bypass with cervical esophagogastric anastomosis. One year later, she presented an esophagocele, treated by esophagectomy by videothoracoscopy in the prone position^7^.

Esophagectomy by videothoracoscopy is a minimally invasive technique and the treatment of choice for many illnesses, from malignant and benign diseases, with low morbidity and mortality, and is a very safe method when performed by experienced surgeons^8^. There were no records found in the literature on esophagoceles treated with videothoracoscopy after caustic ingestion.

Finally, it is important to perform postoperative follow-up in patients with a history of ingestion of caustic products who have undergone esophagogastric bypass, evaluating the possibility of occurrence of esophagoceles.

## CONCLUSIONS

Esophagectomy by videothoracoscopy in a prone position is a feasible and safe option to treat esophagoceles after caustic ingestions.
